# The Synergistic Cooperation between TGF-β and Hypoxia in Cancer and Fibrosis

**DOI:** 10.3390/biom12050635

**Published:** 2022-04-25

**Authors:** Pramod Mallikarjuna, Yang Zhou, Maréne Landström

**Affiliations:** Pathology Section, Department of Medical Biosciences, Umeå University, 901 85 Umeå, Sweden; pramod.mallikarjuna@umu.se (P.M.); yang.zhou@umu.se (Y.Z.)

**Keywords:** cancer, fibrosis, HIF-1α/2α, hypoxia, Smad, TGF-β, TRAF6

## Abstract

Transforming growth factor β (TGF-β) is a multifunctional cytokine regulating homeostasis and immune responses in adult animals and humans. Aberrant and overactive TGF-β signaling promotes cancer initiation and fibrosis through epithelial–mesenchymal transition (EMT), as well as the invasion and metastatic growth of cancer cells. TGF-β is a key factor that is active during hypoxic conditions in cancer and is thereby capable of contributing to angiogenesis in various types of cancer. Another potent role of TGF-β is suppressing immune responses in cancer patients. The strong tumor-promoting effects of TGF-β and its profibrotic effects make it a focus for the development of novel therapeutic strategies against cancer and fibrosis as well as an attractive drug target in combination with immune regulatory checkpoint inhibitors. TGF-β belongs to a family of cytokines that exert their function through signaling via serine/threonine kinase transmembrane receptors to intracellular Smad proteins via the canonical pathway and in combination with co-regulators such as the adaptor protein and E3 ubiquitin ligases TNF receptor-associated factor 4 (TRAF4) and TNF receptor-associated factor 6 (TRAF6) to promote non-canonical pathways. Finally, the outcome of gene transcription initiated by TGF-β is context-dependent and controlled by signals exerted by other growth factors such as EGF and Wnt. Here, we discuss the synergistic cooperation between TGF-β and hypoxia in development, fibrosis and cancer.

## 1. Introduction

Hypoxia is frequently observed during tumor progression, which, in turn, leads to cancer cells starting to secrete immunosuppressive cytokines such as vascular endothelial growth factor (VEGF) and transforming growth factor β (TGF-β) [1,2]. High levels of TGFβ1 as a response to tissue damage have been found to be strongly correlated with the development of connective tissues in the affected organs due to the production of collagen and subsequent fibrosis in tissues such as the kidneys or lungs [3,4]. In this review, we focus on the role of TGF-β in relation to hypoxia, fibrosis and cancer and discuss recent insights into the role of TGF-β signaling and hypoxia in the most common forms of cancer such as prostate, kidney, lung, breast, cervix, gastric, colon, liver and pancreas. To obtain a better understanding of the underlying complex and inter-molecular mechanisms between TGF-β and hypoxia signaling pathways is important, as it could lead to the generation of novel and more specific treatment strategies for patients with cancer and fibrosis. Moreover, we also discuss potential biomarkers and novel therapeutic strategies that might lead to the future development of precision medicine for patients with certain forms of cancer.

TGF-β is a pleiotropic cytokine that structurally belongs to a superfamily that includes activin and bone morphogenic proteins [5,6,7]. TGF-β superfamily signaling is crucial for normal embryogenesis in animals and humans [8,9]. TGF-β signaling is elicited by the three different ligands TGF-β1, TGF-β2 and TGF-β3 (7). In this review we use TGF-β as a common denominator for the ligand, if it has not been stated by the authors of the article we cite, that the response is the result of a specific TGF-β ligand used in their study. In the adult, physiological TGF-β signaling is a safeguard against cancer due to its inhibitory effects on the cell cycle and proliferation in normal epithelial cells [10]. TGF-β also promotes the apoptosis of epithelial cells, thereby acting as a tumor suppressor to prevent the accumulation of mutations in the DNA [11]. Normal physiological TGF-β signaling is also essential for keeping the immune response under strict control to counteract autoimmune responses [12,13]. High levels of TGF-β are found in tumors and in the circulation of cancer patients, which also reflect poor prognosis in several forms of cancer [14,15,16]. Aberrant TGF-β signaling due to mutations or deletions in components of the TGF-β signaling pathway is observed, for instance, in colorectal cancer and pancreatic cancer [17,18,19,20]. The complex and dual role of TGF-β as both a tumor suppressor and tumor promoter [21] underscores the potential risk associated with inhibiting the physiological effects of TGF-β, while there is an urgent need to interfere with its tumor-promoting effects [20,22].

The TGF-β stimulation of epithelial cells leads to their transdifferentiation to acquire a mesenchymal phenotype and to transform them to become mobile and invasive in a process called EMT [23,24]. TGF-β’s effect of promoting EMT is particularly prominent in the presence of the Ras effector RREB [25]. TGF-β mediated extracellular matrix (ECM) synthesis occurs during development and maintenance of mesenchymal tissues as well as in fibrosis [4,26,27,28,29,30]. TGF-β efficiently activates specific transcription programs in both epithelial and mesenchymal cells leading to the accumulation of extracellular matrix proteins, which results in fibrosis in different organs [3,31,32,33,34,35]. We also discuss the role of TGF-β during hypoxia below. 

## 2. TGF-β Signals Are Transduced by Canonical and Non-Canonical Signaling Pathways

**Figure 1:**
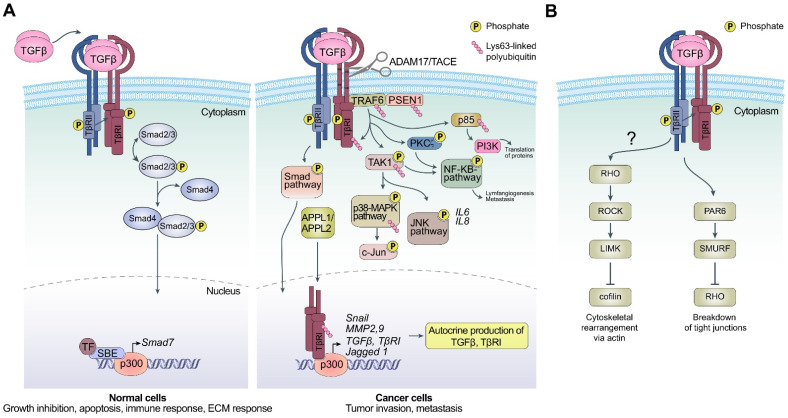
(**A**) A graphical illustration of canonical (left) and non-canonical (right) TGF-β signaling. In canonical TGF-β signaling, TGF-β ligand binds to TGFβRI (TβRI) and TGFβRII (TβRII) receptors, resulting in their activation in a hetero-tetrameric complex. The intracellular Smad2/3 are next activated by phosphorylation, initiated by TGFβRI; pSmad2/3 form a complex with Smad4 and, together with certain transcription factors (TF), activate specific targets genes that contain Smad-binding elements (SBEs), implicated in growth inhibition, apoptosis, ECM synthesis, and immune response. In the non-canonical signaling pathway, TGF-β induces the expression of TRAF6 and ubiquitinates it in a Lys63-dependent manner, promoting its catalytic activity. TRAF6 then activates TACE and PSEN1, resulting in the proteolytic cleavage of TGFβRI, generating the soluble TGFβRI intracellular domain (TGFβRI-ICD/ TβRI-ICD). The endosomal adaptor proteins APPL1/APPL2 and intact microtubules are required for the translocation of TGFβRI-ICD to the nucleus, where it contributes to activating specific target genes. Other modes of non-canonical TGF-β signaling pathways are also shown. Adapted from [43,44,46,47,48,49,50,51,54]. (**B**) TGF-β signaling can also induce EMT through the activation of non-Smad pathways such as Rho GTPase and via the phosphorylation of the polarity protein PAR6, leading to cytoskeletal rearrangements and the breakdown of epithelial cell junctions, respectively. Adapted from [52,53,54,55,56].

The TGF-β signaling pathway is under tight control during embryogenesis and in normal healthy tissues. The TGF-β ligand is kept inactive during protein synthesis and secretion to the extracellular space. The ligand can then be released by proteolytic enzymes such as matrix metallopeptidase 2 (MMP2) and matrix metallopeptidase 9 (MMP9) [36,37]. Integrins and tension in the tissue can release the ligand so that it can bind in a dimeric fashion to the transmembrane TGF-β receptors, which are serine/threonine kinases [12,20,38]. When the ligand binds to the TGF-β type II receptor (TGFβRII), which is constitutively active, it recruits the TGF-β type I receptor (TGFβRI) or ALK5-full length (ALK5-FL), resulting in the formation of a heterotetrameric complex, in which the TGFβRII kinase activity phosphorylates the TGFβRI, which leads to its activation as a kinase. Thereby, the activated TGFβRI can, in turn, phosphorylate the intracellular Smad proteins Smad2 and Smad3 at their C-terminal ends, thereby converting them to become active as transcription factors in a nuclear complex together with Smad4 [5,7,12]. The activity of the nuclear Smad complex for the regulation of gene transcription is highly versatile and dependent on the physiological context (Figure 1A); for excellent reviews for further reading, see [3,5,24,38,39]. *Smad7* is a direct target gene for the TGF-β-induced nuclear Smad complex and was originally classified as an inhibitory Smad [5]. Smad7 is also directly involved in gene transcription in cancer cells, as the Smad7 protein can bind to specific regions in the promoter of *c-Jun* and histone deacetylase 6 (*HDAC6*) in prostate cancer cells, promoting migration and invasion [40]. Nuclear Smad7 was recently shown to regulate gene transcription in mouse embryonic stem cells [41]. The activity of the TGFβ signaling pathways are regulated at several levels, often in negative feedback loops as for Smad7 when by binding to the TGFβRI, Smad7 inhibits TGFβ-Smad canonical signaling [5]. Yet another way to regulate function of TGFβ signaling is exemplified by the fact that TGFβRI undergoes proteolytic cleavage by the activated disintegrin and metalloprotease 17 (ADAM17)/tumor necrosis factor-α-converting enzyme (TACE), and thereby TGF-β can no longer cause growth inhibition in cancer cells [42].

In the non-canonical signaling pathway, TNF receptor-associated factor 6 (TRAF6) has been shown to play important key roles in the diversity of the effects of the TGF-β signaling pathway in cancer cells, leading to either apoptosis or EMT [43,44]. In mammary cancer cells, TNF receptor-associated factor 4 (TRAF4) is amplified and promotes cancer cell invasion and metastasis [45]. Here, we focus on the role of TRAF6 in promoting certain cellular responses in cancer cells (Figure 1A).

TRAF6 binds to TGFβRI via a specific consensus binding site identified in TGFβRI [43]. When the TGF-β ligand binds to the TGFβRII/TGFβRI complex, it results in the formation of a heterotetrameric complex in which the enzymatic properties of TRAF6 become activated. Active TRAF6 thereby promotes the Lys63-linked polyubiquitination of transforming growth factor beta-activated kinase 1 (TAK1), a member of the mitogen activated protein kinase kinase kinase (MAPKKK) family, resulting in its activation as a kinase. The subsequent activation of the downstream p38 MAPK/JNK pathways as well as the nuclear factor kappa B (NF-κB) pathways leads to EMT, apoptosis, migration, invasion and inflammatory responses through activator protein-1 (c-Jun/c-Fos) and p65 transcription factors, respectively [43,46]. In cancer cells, TRAF6 also engages and activates proteolytic enzymes such as ADAM17/TACE and presenilin-1 (PSEN1 or PS1), which leads to the generation and release of the TGFβRI intracellular domain (TGFβRI-ICD) [47,48]. The Lys63-linked polyubiquitination of TGFβRI on Lys178 by TRAF6, together with its binding to the early endosomal proteins, adaptor protein, phosphotyrosine interacting with PH domain and leucine zipper 1 (APPL1)/(APPL2), and microtubule system, transfer the soluble TGFβRI-ICD to the nucleus, where it binds to the transcriptional co-regulator p300 [47,49,50]. Within the nucleus, the TGFβRI-ICD promotes the transcription of TGF-β and TGFβRI itself, as well as pro-invasive genes such as *SNAIL1* and *MMPs*, further promoting the activity of TGF-β in this positive forward signaling loop, leading to enhanced EMT, conferring migratory and invasive capabilities on cancer cells. TGF-β induces the sumoylation of K234 on SNAIL1, promoting EMT [51]. TGF-β signaling can also induce EMT through the activation of non-Smad pathways such as Rho GTPase and via the phosphorylation of the polarity protein PAR6, leading to cytoskeletal rearrangements and the breakdown of epithelial cell junctions [52,53] (Figure 1B).

The generation of nuclear TGFβRI-ICD or ALK5-ICD is also dependent on an atypical protein kinase C (aPKC) called protein kinase C zeta (PKCζ) [47]. The knockdown of PKCζ in three different prostate cancer cell lines in vivo leads to a reduction in tumor growth and metastasis [57]. The role of TRAF6 as an oncogene in cancer has recently emerged, as TRAF6 is amplified in several types of cancer, such as lung carcinoma [58], advanced prostate cancer [59], gastric carcinoma [60] and bladder cancer [61], and promotes angiogenesis and glioblastoma progression [62,63]. For further reading about the oncogenic role of TRAF6 in cancer, see a recent review [64].

The capability of TGFβRI-ICD (or ALK5-ICD) and function of the nuclear TGFβRI-ICD to promote the invasion of clear-cell renal carcinoma cells (ccRCCs) in vitro has also been demonstrated. Moreover, high levels TGFβRI-ICD in clinical ccRCC tissues derived from patients have been shown to correlate with poor prognosis [65,66]. Interestingly the TGFβRI was also found to be in a complex together with HIF1-α/HIF2-α in clear cell renal cell carcinoma (ccRCC)s in vitro, suggesting a role for TGFβRI in hypoxia [67], consistent with previous reports about a functional role of TGF-β in promoting angiogenesis [68,69,70]. 

## 3. Hypoxia and Von Hippel–Lindau (VHL)

**Figure 2:**
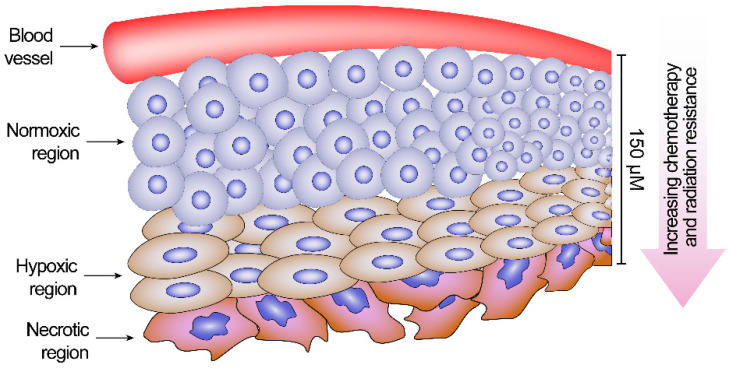
A graphical illustration of varied regions in solid tumors. The tumor region close to the blood vessel contains oxygenated cells, followed by a region of hypoxic cells and necrotic cells. Adapted from [72,73].

Hypoxia is a condition where the levels of oxygen are severely reduced, often leading to pathogenesis. Normal oxygen levels are called normoxia, and extreme hypoxia or total oxygen deprivation is called anoxia (Figure 2) [71]. 

**Figure 3:**
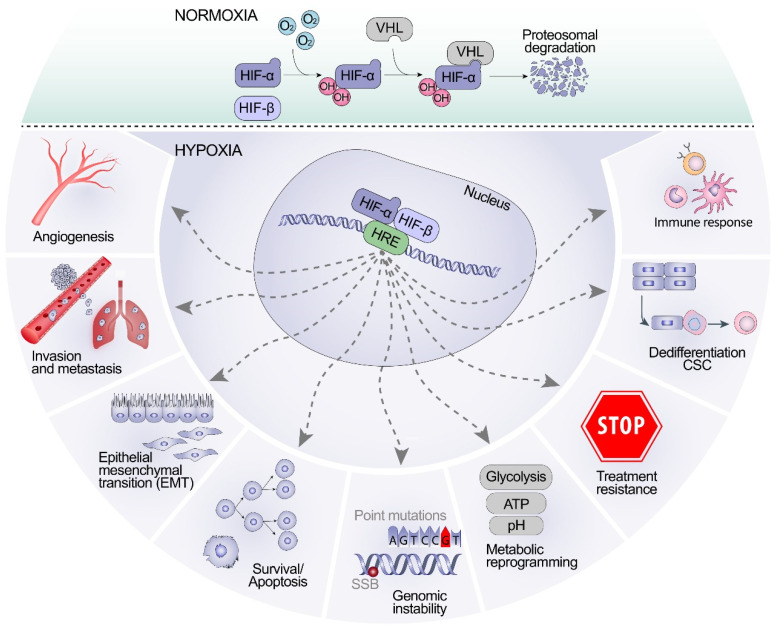
A graphical illustration of normoxia and hypoxia. HIF-α is degraded by VHL under normoxic conditions. Under hypoxic conditions, HIF-α is stabilized and induces the expression of target genes that are associated with tumor promotion. SSB (single-strand break), CSC (cancer stem cell). Adapted from [79].

Von Hippel–Lindau protein (pVHL) tumor suppressor is an E3 ubiquitin ligase and it works in an oxygen-dependent manner. In the presence of oxygen, pVHL targets HIF-α for ubiquitination and subsequent proteasomal degradation through the activity of HIF-α associated prolyl hydroxylase (PHD) family members [73]. However, hypoxia or an irregularity in *VHL* renders pVHL ineffective; this leads to the stabilization of HIF-α, which activates the expression of several target genes that facilitate tumor progression. pVHL is a well-established tumor suppressor gene, and importantly, it regulates the expression of hypoxia-inducible factors (HIFs). HIF-1α and HIF-2α are associated with the induced expression of genes associated with tumor promotion [73,74,75,76,77] (Figure 3). Interestingly, pVHL has recently been demonstrated to control stability of Smad3 in normal epithelial cells by marking Smad3 for degradation both in mammalian epithelial cells and in the Drosophila wing during development; pVHL can directly control the transcriptional outcome of the TGF-β-Smad3 canonical signaling pathway [78]. Further studies will elucidate the impact of these findings on development of cancer and fibrosis in relation to TGF-β canonical and non-canonical signaling pathways but are consistent with the previous identification that also TGFβRI is a target for pVHL in ccRCC cells [66].

## 4. Synergistic Cooperation between TGF-β and Hypoxia in Fibrosis

Fibrosis occurs in several organs as the outcome of wound healing following recurrent tissue injury. It is defined by the dysregulated production and excessive accumulation of collagen-rich ECM, as well as the replacement of normal functional tissue with fibrotic tissue [80]. This process can affect the function of vital organs such as the heart, lungs, liver and kidneys and is involved in the pathological progression of different cancer diseases, including clear-cell renal carcinoma [31,81,82]. Hypoxia and TGF-β signaling synergize in the regulation of fibrosis. In this review we used the following terms as defined below: Synergistic cooperation is meaning effects greater than adding the sum of two individual effects. Additive effects refer to the same as the sum of the two. A feedback loop between hypoxia and TGF-β refers to connections between TGF-β and hypoxia in a linear pathway (e.g., when the hypoxia effect is mediated by TGF-β or the other way around). 

TGF-β signaling promotes the activation of fibroblasts and the remodeling of ECM. Generally, local resident fibroblasts differentiate into myofibroblasts that express α-smooth muscle actin (α-SMA), type I collagen, fibronectin and other ECM components [83]. Interestingly, other mesenchymal cells can also be activated into a myofibroblast phenotype. In the kidney, for example, TGF-β-Smad signaling transforms mesangial cells into fibroblasts, a process that can be repressed by AMP-activated protein kinase (AMPK) [84]. TGF-β signals via activated Smad proteins promotes the transcription of the type I collagen genes COL1A1 and COL1A2, a process that is partially abrogated by p53 [85]. These mRNAs are targeted by several microRNAs, including miR-29, miR-96, miR-130b and miR-326, whose expression is repressed by TGF-β, promoting collagen protein expression [31]. The proper folding of collagen chains in the endoplasmic reticulum (ER), trafficking through the Golgi and secretion require the chaperones Hsp47p and Fkbp10p, the expression of which is induced by TGF-β [86,87]. The extracellular processing of collagen is also TGF-β-regulated. For example, the TGF-β-induced expression of Loxl4p, a lysyl oxidase-like protein, contributes significantly to type I collagen crosslinking. TGF-β/hypoxia-induced Loxl2p expression promotes collagen crosslinking, contributing to ECM remodeling in fibrosis and metastasis [88,89,90]. It has been observed in dermal fibroblasts that hypoxia upregulates TGF-β/Smad signaling via HIF-1α, promoting the expression and deposition of type I collagen [91,92,93]. Besides collagen, TGF-β induces the expression of genes encoding α-SMA and fibronectin, as well as many other ECM components, including plasminogen activator inhibitor 1 (PAI-1) and tissue inhibitor of metalloproteinase (TIMP). Collectively, TGF-β regulates ECM remodeling by promoting the activation of myofibroblasts and the accumulation of ECM components, and hypoxia cooperates with TGF-β signaling to regulate the synthesis and deposition of ECM [3,31].

TGF-β signaling promotes fibrosis progression by inducing EMT and endothelial–mesenchymal transition (EndMT) in different organs. Epithelial cells lose their original properties such as apical–basal polarity and cell–cell adhesion and acquire mesenchymal features such as anterior–posterior polarity and a cytoskeleton that favors migration and invasion. The expression of epithelial markers such as E-cadherin is repressed, while the expression of mesenchymal markers such as N-cadherin, α-SMA, fibronectin and vimentin is induced. EndMT is a similar process that occurs in endothelial cells, and TGF-β is recognized as the most important inducer of EMT/EndMT [94]. TGF-β signaling can, through the Smad-dependent pathway, directly activate the expression of EMT transcription factors such as SNAIL, TWIST and Zinc Finger E-Box Binding Homeobox (ZEB) and then cooperate with them to regulate the expression of target genes. TGF-β signaling can also induce EMT through the activation of non-Smad pathways such as Rho GTPase and via the phosphorylated polarity protein PAR6, promoting cytoskeletal rearrangements and the breakdown of epithelial cell junctions, respectively (Figure 1B) [52,53,95]. Tubular epithelial cells in the kidney, epicardial cells, hepatocytes, cholangiocytes and alveolar epithelial cells can undergo EMT to generate fibroblasts in response to injury or stress [96,97,98]. Hypoxia has been demonstrated to synergize with TGF-β signaling during the induction of EMT, to different degrees in different models. In alveolar epithelial cells, hypoxia induces EMT in a TGF-β-dependent manner [99]. In a renal fibrosis model, TGF-β signaling participates in, but is not required for, hypoxia-induced EMT and fibrogenesis [100]. In hypoxic hepatocytes, hypoxia stimulates hepatocyte EMT by a HIF and TGF-β-dependent mechanism, where latent TGF-β1 is activated downstream of HIF signaling [101].

TGF-β signaling exerts multiple effects on inflammation and fibrosis. Fibrosis is regarded as the final state of many chronic inflammatory diseases, while inflammation is recognized as an early stage of fibrosis [96]. Macrophages, either resident or differentiated from circulating monocytes, are recruited to the site of tissue injury or inflammation. They are activated as pro-inflammatory M1 macrophages at the early stage of inflammation, while the anti-inflammatory M2 macrophages later become dominant. Several cytokines, including TGF-β, promote the activation of M2 macrophages, and the activated M2 macrophages, in turn, produce profibrotic factors, including TGF-β [102,103]. TGF-β is generally recognized as an anti-inflammatory cytokine, as TGF-β1-null mice show excessive inflammatory responses [104]. This is consistent with the role of TGF-β in promoting the generation of immunosuppressive T-regulatory cells [105]. In addition to the production of TGF-β, macrophages also contribute to the myofibroblast pool via TGF-β/Smad-induced macrophage–myofibroblast transition in a renal fibrosis model [106], contributing to the myofibroblast population and fibrogenesis. Collectively, TGF-β plays an important regulatory role during the inflammation–fibrosis transition.

**Table 1:** Tabulation of selected genes and proteins in different forms of cancer regulated by synergistic cooperation between TGF-β and hypoxia; and specific characteristics associated with them. See also Figure 4.

Cancer Type	Gene/Protein	Upregulation/ Downregulation	Cancer Progression Hallmark	Reference
Kidney cancer	CA9	Upregulation	pH control	[67]
	GLUT1	Upregulation	Glucose transport	[67]
Prostate cancer	VEGF	Upregulation	Angiogenesis	[107]
	CXCL13	Upregulation	Metastasis	[108]
Pancreatic cancer	Fibulin-5	Upregulation	Protection from apoptosis	[109]
	PKCα	Upregulation	EMT	[110]
	Nestin	Upregulation	EMT and cell migration	[111]
	NOX4	Upregulation	Metastasis	[112]
Lung cancer	NRF2	Upregulation	Radio resistance	[113]
	EGFR	Upregulation	Radio resistance	[113]
	CD39 and CD73	Upregulation	Immune regulation	[114]
Liver cancer	CA9	Upregulation	pH control	[115]
Gastric cancer	Treg	Upregulation	Immune regulation	[116]
	Foxp3	Upregulation	Immune regulation	[116]
Colorectal cancer	DDB2	Downregulation	Protects against EMT	[117]
Breast cancer	VEGF	Upregulation	Angiogenesis	[2]
	CXCR4	Upregulation	Metastasis	[2]
	ITGB3	Upregulation	Metastasis	[118]
	BRMS1	Downregulation	Protect against EMT	[119]
	miR-191	Upregulation	Cancer cell migration	[120]
Cervical cancer	PLOD2	Upregulation	Collagen regulation/EMT	[121]

**Figure 4:**
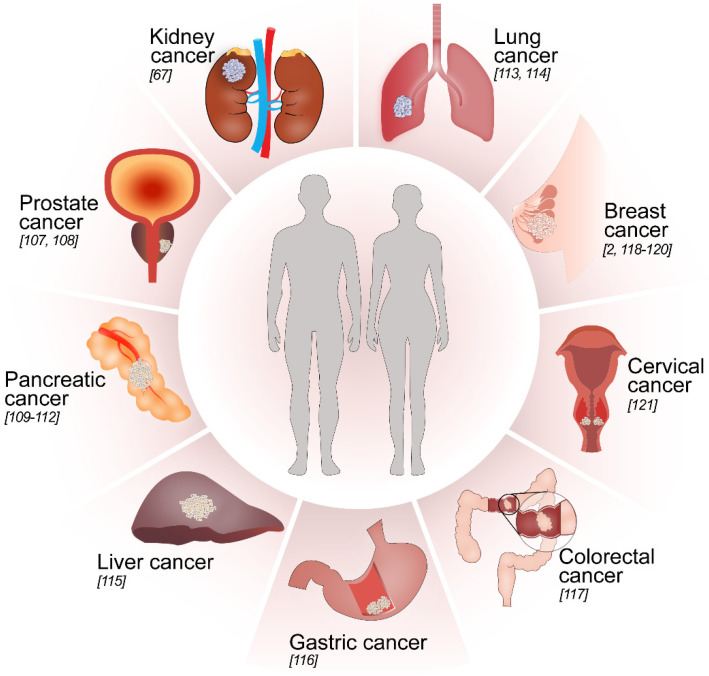
A graphical illustration of several organs where TGF-β and hypoxia contribute to tumor aggressiveness; included are references to studies that have investigated synergistic cooperation between TGF-β and hypoxia in respective cancer types. See also Table 1.

## 5. Synergistic Cooperation between TGF-β and Hypoxia in Cancer

### 5.1. Kidney Cancer

**Figure 5:**
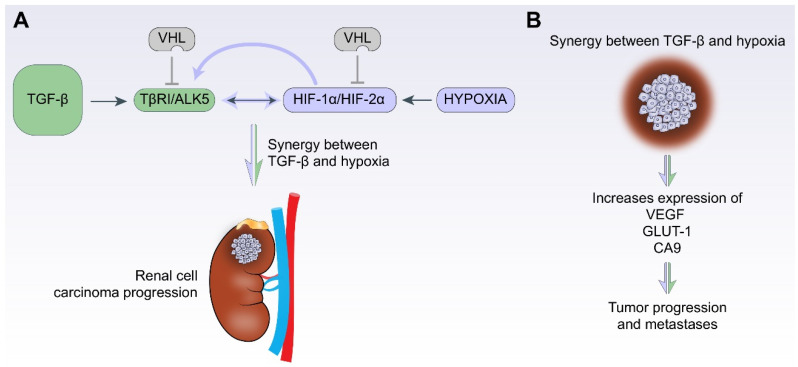
A graphical illustration of crosstalk between TGF-β and hypoxia in renal cell carcinoma and solid tumors. TGF-β and hypoxia pathways promote tumor progression in renal cell carcinoma (**A**) and other solid tumors (**B**) through TGFβRI (TβRI) and HIF-1α/2α and enhances expression of VEGF, CA9 and GLUT1 [2,67,73,123,124]. Adapted from [67].

Kidney cancer or renal cell carcinoma (RCC) constitutes about 2.2% of all the cancers diagnosed worldwide [122]. We previously investigated the roles of TGF-β [65,66,67] and hypoxia [67] in renal cell carcinoma and mainly focused on the ccRCC subtype [65,67]. Canonical (Smads) and non-canonical (via TGFβRI-ICD or ALK5-ICD) TGF-β signaling was significantly positively associated with tumor progression and poor patient survival in ccRCC [65]. As discussed previously in the introduction section, VHL is a common ubiquitin ligase for both HIF-αs [77] and TGFβRI/ALK5 [66]; its absence leads to enhanced crosstalk between the hypoxic and TGF-β pathways, consistent with the recent identification of Smad3 as a target for pVHL, as described above [78]. Our studies on ccRCC clinical samples show a positive correlation between components of TGF-β signaling and hypoxia [67] (see Figure 4). RCC cell lines overexpressing TGFβRI/ALK5 and treated with TGF-β showed an increase in both TGF-β and hypoxia pathway targets. The absence of VHL enhances this increase in a manner dependent on the kinase activity of TGFβRI/ALK5. Hypoxia treatment also increases the levels of TGF-β signaling components irrespective of the status of VHL. As TGF-β and HIFs stimulate the expression of each other, we explored a possible link between the upstream components of these two pathways with the aim to identify the nexus points. Immuno-precipitation and a proximity ligation assay performed in RCC cell lines revealed a physical interaction between TGFβRI/ALK5 and HIF-1α/2α (Figure 5) [67]. Another study investigated expression analyses in RCC cell lines and revealed an extensive overlap between the TGF-β response and genes regulated by the HIF [123].

These studies [67,123] show that synergistic interaction between TGF-β and hypoxia contributes to ccRCC disease progression.

### 5.2. Prostate Cancer

Patients with local non-metastasized prostate cancer have excellent survival rates. Contrastingly, metastasized prostate cancer has a high mortality rate and is one of the leading causes of cancer-related death in men in several countries [125]. A necessary event in metastasis is angiogenesis, regulated by vascular endothelial growth factor (VEGF) [126], a target of hypoxia [127,128]; TGF-β also increases the expression of VEGF [129]. VEGF is the major angiogenic cytokine expressed in normal human prostate epithelial cells [130], and its overexpression is associated with an aggressive and invasive tumor phenotype [131]. Hypoxia and TGF-β are major factors that increase VEGF production and thereby increase angiogenesis, both independently and in cooperation [132,133,134]. Hypoxia enhances TGF-β expression, and this produces a feedback loop that increases VEGFA production [107]. It has also been shown that TGF-β is an autocrine regulator of hypoxia-mediated VEGFA_165_ isoform secretion in prostate cancer cells [107] (see Figure 4). As discussed in this review, TGF-β treatment also increases the levels of VEGF in gastric cancer [135] and breast cancer [136]. A study reported that TGF-β differentially influenced the expression of VEGFA in kidney cancer cell lines [137]. TGF-β did not significantly influence expression of VEGFA in OS-RC-2 cell lines, but slightly increased VEGFA in Caki-1 cell lines [137]. In contrast, TGF-β has been reported to suppress VEGFA-mediated colon cancer metastasis [138]. The role of TGF-β in these contexts may be cell-line-specific and organ-specific.

Another mode by which hypoxia and TGF-β cooperate in promoting prostate cancer aggressiveness is the activation of myofibroblasts through chemokine (C-X-C motif) ligand 13 (CXCL13) expression [108]. CXCL13 is a B-cell chemoattractant [139]. Its expression in prostate cancer is correlated with disease severity [140]. Tumor myofibroblasts express CXCL13, and TGF-β induced by hypoxia drives the expression of CXCL13, leading to accelerated malignant progression and castration-resistant prostate cancer development [108] (see Figure 4). Hypoxia and TGF-β stimulate each other to fuel these loops and contribute to aggressive and invasive prostate cancer. 

In accordance with a study on kidney cancer [67], which showed that TGF-β increases HIF-1α and HIF-2α under normoxic conditions, a study on prostate cancer cell lines revealed that TGF-β induces HIF-1α, HIF-2α and VEGF production under normoxic conditions. This is brought about by the interaction between Smad3 and HIF-2a, in a SBE2 and hypoxia response elements (HRE) dependent manner [141]. Thus, TGF-β can activate HIF target genes in normoxic conditions and even more in hypoxic environments, so that they both contribute to further fuel these synergistic loops and promote the development of aggressive and invasive prostate cancer.

### 5.3. Pancreatic Cancer

Pancreatic cancer patients have extremely poor survival rates, and this trend has not changed significantly during the past few decades [142]. Hypoxia and TGF-β are both increased in pancreatic cancer [143,144]. 

Fibulin-5 (Fbln5) is a matricellular protein that reduces microenvironmental reactive oxygen species (ROS) and protects pancreatic tumor cells [145]. Hypoxia induces Fbln5 in endothelial cells and makes these cells resistant to hypoxia-mediated apoptosis [146]. TGF-β also induces Fbln5 in vitro [147]. A study has revealed that hypoxia induces Fbln5 in a TGF-βRI/ALK5-dependent and Smad4-independent manner, and hypoxia and TGF-β increase the expression of Fbln5 in pancreatic cancer [109] (see Figure 4). 

Increased protein kinase C-α (PKCα) levels are an unfavorable indicator for patient survival in pancreatic cancer [148] and are associated with EMT [149]. The activation of PKCα by hypoxia and TGF-β leads to aggressive pancreatic cancer [110] (see Figure 4), and TGF-β also plays a role in PKCα-induced drug resistance in pancreatic cancer [150]. PKCα also regulates HIF-1α [151] and TGF-β [152].

Nestin is a functional stem-cell marker. Recent investigations have revealed that nestin is also a cancer stem-cell marker, and it contributes to pancreatic cancer development [153]. Hypoxia increases TGF-β levels in pancreatic cancer cells, and TGF-β in a Smad4-dependent manner increases nestin expression, resulting in increased tumor-cell migration and EMT [111] (see Figure 4). 

NADPH oxidase 4 (NOX4) is an enzyme that increases the invasiveness and metastasis of pancreatic cancer cells. Hypoxia induces NOX4 activation in a TGF-β-dependent manner [112] (see Figure 4). 

### 5.4. Lung Cancer

Lung cancer is a common cancer, accounting for more than 11% of all cancers diagnosed worldwide [154]. NF-E2 p45-related factor 2 (NRF2) is a redox-sensitive transcription factor highly expressed in lung cancer [155]. Epidermal growth factor receptor (EGFR) is a well-known oncogenic tyrosine kinase activated in lung cancer [156]. Combined TGF-β and hypoxia treatment in lung cancer cells significantly increases the expression and translocation of NRF2 into the nucleus and the crosstalk between NRF2 and EGFR. Furthermore, the combined treatment imparted radioresistance to lung cancer cells [113] (see Figure 4). NOX4 has been described before in the pancreatic cancer section, and it is regulated by hypoxia and TGF-β [112]; NOX4 regulates the expression of NRF2 [157].

Myeloid-derived suppressor cells, or MDSCs, are regulators of the immune system [158]. Cluster of differentiation 39 (CD39) and cluster of differentiation 73 (CD73) are ectonucleotidases, and MDSCs expressing CD39 and CD73 are increased in non-small-cell lung cancer [114]. TGF-β and HIF-1α are capable of inducing CD39^+^/CD73^+^ myeloid cells [159,160]. A study has shown that TGF-β induces the expression of CD39 and CD73 in MDSCs by activating the mechanistic target of rapamycin (mTOR)–HIF-1α pathway [114] (see Figure 4). 

Another study revealed interesting findings with experiments on Lewis lung carcinoma cells (LLCs) [161]. Short-term exposure to hypoxia had an inhibitory effect on the transcriptional activity of TGF-β in LLCs compared to cells under normoxia. However, when the cells were subjected to hypoxic conditions for a significantly longer time, the TGF-β-mediated responses via Smad phosphorylation were enhanced. Furthermore, hypoxia increased Smad2/Smad4 interaction, *TGF-β* mRNA expression, and *Smad7* and *SNAIL* gene expression. Importantly, TGFβRI/ALK5 expression was also increased by hypoxia, without TGF-β treatment. It is notable that TGF-β treatment did not affect HIF-1α signaling [161]. Similar alterations are observed in ccRCC [67] and prostate cancer [141]. Further, in these two types of cancers, TGF-β increased the expression of HIF-1α and HIF-2α as well [67,141]. A study on lung cancer cells showed that stabilized HIF-1α had an inhibitory effect on TGF-β-induced matrix production, in part, through protein-phosphatase activity [162]. This discrepancy may be due to the different cell lines used and the different time points at which these cell lines were subjected to hypoxia. 

### 5.5. Liver Cancer

Liver cancer is the sixth most common cancer globally and has the fourth highest mortality rate worldwide. Hepatocellular carcinoma (HCC) is the primary liver malignancy [163]. Even though the diagnosis and treatment of HCC have improved, the 5-year survival rate is only 18%, making it the second lowest among cancers [164,165,166]. In HCC, as in other cancers, EMT plays a crucial role in the generation of metastasis [167]. Both TGF-β and hypoxia contribute to EMT [168]. It is also interesting to note that HCC is one of the most hypoxic tumors [71].

Levels of TGF-β and HIF-1α are both increased in HCC and are positively correlated with each other and contribute to poor prognosis [166]. A feed-forward loop was activated due to TGF-β and hypoxia increasing each other’s expression, and this loop facilitated tumor progression [166]. Like this observation in HCC cell lines, the ability of hypoxia and TGF-β to stimulate the components of each other’s pathway has been documented in ccRCC [67], prostate cancer [141] and lung cancer [161]. 

Carbonic anhydrase 9 (CA9) is a transmembrane protein that is often found to be associated with aggressive cancers [169]. CA9 is an endogenous marker for hypoxic cells [170] and regulates pH in hypoxic cancer cells [171]. TGF-β, under hypoxic conditions, can increase the levels of CA9 in the Hep3B liver cancer cell line. This increase is dependent on phosphoinositide 3-kinases (PI3K) and the MAPK pathway [115] (see Figure 4). A different study on the same cell line reported that extreme hypoxia caused the upregulation of HIF-1α and CA9; TGF-β treatment along with extreme hypoxia almost doubled the expression of HIF-1α and CA9 [172]. A similar effect of TGF-β and hypoxia on the expression of CA9 is observed in ccRCC cell lines [67], underscoring the synergistic function of these two factors in tumor progression. 

### 5.6. Gastric Cancer

Gastric cancer is the fifth most common cancer and the third most lethal [173]. A study used several gastric cell lines (OCUM-2MD3, OCUM-12 and OCUM-2M) and reported interesting findings concerning their response to hypoxia and TGF-β [174]. TGF-β and hypoxia rendered a spindle shaped morphology in these cell lines. Hypoxia differentially altered the expression of different component of TGF-β signaling and its targets associated with mesenchymal phenotype [174]. 

Regulatory T-cells or Tregs have an inhibitory effect on immune cells [175] and are upregulated in several cancers, including gastric cancer [176]. A study investigated the levels of HIF-1α and forkhead box protein P3 (Foxp3), a protein involved in immune suppression implicating Tregs [116,175,177]. Immunohistochemistry of tumor tissues revealed a strong co-expression between HIF-1α, Foxp3 and TGF-β. Foxp3-positive cells mainly assemble in hypoxic tumor regions, where cells also express high levels of HIF-1α and TGF-β. Hypoxia increased the expression of HIF-1α, Foxp3 and TGF-β in gastric cancer cell lines [116] (see Figure 4). 

A study subjected HGC27 and MGC803 gastric cancer cell lines to normoxia, hypoxia and severe hypoxia to study the effects on EMT [178]. Under severe hypoxic conditions, the levels of proteins that are involved in EMT were significantly increased, and increased TGF-β levels and enhanced autocrine TGF-β production in gastric cancer cells and activated Smad2 and Smad3, and PI3K/Akt signaling was observed, which further augmented EMT [178]. 

All these studies on gastric cancer [116,174,178] are in accordance with studies exploring the relation between TGF-β and hypoxia in ccRCC [67], prostate cancer [141], lung cancer [161] and liver cancer [166], mentioned in their respective sections. 

### 5.7. Colorectal Cancer

Colorectal cancer (CRC) accounts for 9% of all cancers diagnosed worldwide [179]. 

Damaged DNA-binding protein (DDB)-2 is involved in remodeling damaged chromatin in the early steps of nucleotide-excision repair [180], its deficiency increases the invasiveness and tumorigenicity of colon cancer cells, and its absence contributes to EMT [117]. The authors examined if DDB2 inhibited EMT induced by TGF-β and hypoxia. Colorectal cancer cells overexpressing *DDB2* were subjected to TGF-β treatment and hypoxia. E-cadherin was reduced due to TGF-β or hypoxia treatment; however, in the presence of *DDB2*, this reduction was not very significant [117] (see Figure 4), suggesting that DDB-2 counteracted TGF-β-induced EMT. 

Sciellin or SCEL, an arterial intima-enriched protein that contributes to the stress properties of the stratified epithelium [181,182,183], was found to be highly expressed in CRC cell lines that had liver metastasis, played a significant role in invasion and migration, and correlated with CRC malignancy. Interestingly, SCEL participated in mesenchymal-to-epithelial transition (MET) [183]. It has been reported that, for clonal outgrowth at metastatic sites, MET is necessary for the proliferation and differentiation of cancer cells [184]. CRC cell lines were treated with TGF-β or subjected to hypoxia. These treatments induced the expression of mesenchymal markers such as vimentin and reduced the levels of epithelial markers such as E-cadherin. Both TGF-β and hypoxia inhibited SCEL levels. However, when cells were transferred back to normoxia, the expression of these markers was reversed [183]. 

In CRC, TGF-β and hypoxia cooperate to modulate EMT [117,183]. These two pathways also modulate EMT in ccRCC [67], pancreatic [111], liver [168] and gastric cancers [178].

### 5.8. Breast Cancer

Breast cancer is the most common form of cancer in women in several countries [154]. A study investigated the roles of hypoxia and TGF-β in promoting bone metastasis in breast cancer [2]. The overexpression and co-expression of HIF-1α and Smads increases the expression of VEGF and C-X-C chemokine receptor type 4 (CXCR4) promoter activity in response to hypoxia and TGF-β. TGF-β and hypoxia synergize to regulate VEGF and CXCR4 transcription, and inhibition of HIF-1a or TGF-β, or TGFβRI/ALK5, resulted in reduced bone metastasis in a mouse model [2] (see Figure 4). 

Breast cancer cell cultures in mesenchymal stem cells (MSC)-conditioned medium under hypoxia showed higher growth rates, increased cell migration, EMT, and phosphorylation of Smad2. TGFβRI-inhibitor treatment diminished these tumor-promoting properties in cells. The study further confirmed that hypoxia upregulated TGF-β expression in MSCs and had a positive correlation with HIF-1α and TGF-β. HIF-1α also drives TGF-β expression by regulating TGF-β promoter activity [185]. 

Integrin beta 3 (ITGB3) is a member of the integrin family, and it is associated with malignant tumor progression and reprogramming the tumor microenvironment [186]. A study investigated if ITGB3 modulated the TGF-β pathway under hypoxic conditions in breast cancer cell lines [118] (see Figure 4). Silencing ITGB3 reduced TGF-β-induced cell migration and SNAIL expression, especially under hypoxia. Smad phosphorylation was also not observed in cells with silenced ITGB3, as ITGB3 may interact with TGF-β receptors in the early steps of the signaling pathway [118,187].

Breast cancer metastasis suppressor 1 (BRMS1) is a relatively novel metastasis suppressor protein [119,188]. BRMS1 significantly reduced the TGF-β-induced expression of SNAIL, TWIST1 and HIF-1α, and both cell invasion and EMT [119]. BRMS1 restored the E-cadherin levels downregulated by TGF-β in a HIF-1α-dependent manner. Furthermore, BRMS1 inhibited the TGF-β-induced translocation of NF-ĸB subunits [119] (see Figure 4). 

microRNA-191 (miR-191) is a hypoxia-regulated miRNA that contributes to aggressive breast cancer. The hypoxia-induced regulation of miR-191 is dependent on HIF, and miR-191 induces TGF-β expression in breast cancer. The TGF-β induced by miR-191 is involved in breast cancer cell migration. Therapy against miR-191 reduced the volume in breast cancer cell line 3D tumor spheroids [120] (see Figure 4). 

Like other organ cancers discussed in previous sections, TGF-β and hypoxia affect the cancer hallmarks such as angiogenesis, EMT and metastasis to promote breast cancer. 

### 5.9. Other Cancers and Cancer Cell Lines

Procollagen-lysine,2-oxoglutarate 5-dioxygenase 2 (PLOD2) is an intracellular enzyme that primarily initiates the lysine hydroxylation of collagen molecules, migration and invasion. In cervical cancer, PLOD2 is expressed in response to TGF-β and hypoxia. Subjecting cervical cancer cell lines to hypoxia increased the levels of HIF-1α and PLOD2, and the migration and invasion of cells mediated by PLOD2 were dependent on HIF-1α. Similarly, upon treatment with TGF-β, the levels of PLOD2 were increased, and EMT was induced, as indicated by the marker expression [121] (see Figure 4). 

TRAF6, a component of non-canonical TGF-β signaling, shows several functional associations with HIF-1α, as demonstrated in colon cancer and cervical cancer cells. When these cells were subjected to hypoxia, it was observed that TRAF6 upregulated the expression of HIF-1α at the post-transcriptional level. Knocking down or inhibiting TRAF6 reduced the levels of HIF-1α and its targets. It is interesting to note that TRAF6 increased the expression of HIF-1α even under normoxic conditions. Furthermore, TRAF6 interacts with HIF-1α and ubiquitinates it in a K63-dependent manner [62]. 

Prostate transmembrane protein, androgen induced 1 (PMEPA1) is a hypoxia-responsive gene. The levels of PMEPA1 were increased by exposure to hypoxia in colon cancer cells and pancreatic cancer cells. Co-treatment with TGF-β along with hypoxia remarkably increased PMEPA1 levels, indicating synergistic cooperation between the two pathways. Enrichment analysis also revealed that TGF-β and hypoxia genes were associated with PMEPA1-correlated genes [189]. Interestingly, a paper has revealed that PMEPA1 inhibits TGF-β signaling via a negative feedback loop; therefore, in some cases, hypoxia through PMEPA1 may provide a negative feedback loop to TGF-β signaling [190]. Further, a study in lung cancer cells reported that TGF-β increases PMEPA1 expression, and it participates in negative feedback control of the duration and intensity of TGF-β⁄Smad signaling [191].

C-X-C motif chemokine 12 (Cxcl12) is a chemokine that is upregulated by hypoxia and TGF-β in hepatocytes. The increment in Cxcl12 in mouse hepatocytes was dependent on HIF-1α and TGF-β [192]. NOD-like receptor family, pyrin domain-containing 3 (NLRP3), is associated with inflammasomes. TGF-β and hypoxia upregulate the expression of NLRP3 in lung cancer cells [193]. TGF-β and hypoxia upregulated EMT in ovarian cancer cells and prostate cancer cells [194]. Multiple myeloma cells are hypoxic in the bone marrow and possess stem-cell-like characteristics. TGF-β signaling contributes to the stemness in multiple myeloma cells [195]. 

Several cell lines were used to study the synergistic action of TGF-β and hypoxia on VEGF gene expression [134]. Hypoxia increased the expression of VEGF, as did TGF-β to a lesser extent. However, when both TGF-β and hypoxia were combined, the increase in VEGF was significantly higher. This increase in expression occurred at the transcriptional level, at which the synergistic cooperation is mediated. HIF-1α and Smads cooperate and bind to the promoter region of *VEGF*, thereby increasing its expression [134]. 

TGF-β stimulation of liver cancer and fibrosarcoma cells leads to nuclear accumulation of HIF-1α, which efficiently causes the transactivation of HRE. As TGF-β also inhibits PHD2 expression through the Smad signaling pathway, this confers stability to HIF-1α and partly contributes to VEGF expression [196]. The effect of TGF-β and hypoxia on VEGF in other forms of cancers has been discussed before in the sections on prostate cancer and kidney cancer. 

An interesting paper has delved into the inhibitory Smad, Smad7 and its relationship with hypoxia [197]. Hypoxia increased the levels of *Smad7* in several cell types. TGF-β and hypoxia cooperated with each other to increase the expression of Smad7. The expression of Smad7 was increased in the absence of pVHL in RCC cell lines. The study suggests that hypoxia-activated Smad7 inhibits TGF-β signaling. Interestingly, the study also suggests that hypoxic conditions render Smad7 a tumor promoter, even though it is a tumor suppressor under normoxic conditions [197].

Recently, it has been shown that both HIF-1α and extracellular adenosine can counteract tumor-reactive T-cells via A2A/A2B adenosine receptors. When cancer cells and tumors are exposed to hypoxia, HIF-1α is stabilized, which subsequently leads to the increased transcription of adenosine-generating ectoenzymes. These gene promoters contain HREs. Adenosine binds to cAMP-elevating A2AR/A2BR on the surface of T-cells, initiating protein-kinase-A-mediated (PKA-mediated) signaling cascades leading to the inhibition of T-cell effector functions and immunosuppressive transcription via cAMP response elements. It is possible that HIF-1α acts in synergy with cAMP signaling by inhibiting T-cell effector functions and, thereby, promoting the immunosuppressive transcription of TGF-β via HREs [198,199].

## 6. Conclusions

Key regulatory factors for tumor progression, fibrosis and hypoxia are the multifunctional cytokine TGF-β and the transcriptional targets for HIFs. Increased levels of HIFs are induced by intratumoral hypoxia, increased expression of oncogenes or loss of expression of tumor suppressors due to genetic alterations as well as increased levels of growth factors. In turn, and as a result of HIF’s transcriptional activity, an increased expression of growth factors will be produced which further fuel tumor growth [73]. A number of therapeutic agents targeting oncogenic growth factor signaling pathways regulated by HIF-1 have been developed during the last decades and several of them have been demonstrated to exert inhibitory effects on tumor growth [200,201]. 

To mention one example of a successful drug is Trastuzumab (Herceptin), an ERBB2/HER2 receptor tyrosine kinase inhibitor frequently used for treatment of breast carcinoma [202]. Another example of a successful cancer drug is Imatinib (Glivec) which targets BCR–ABL and platelet-derived growth factor receptor PDGFR tyrosine kinases which are driving hematopoietic malignancies and gastrointestinal stromal tumors [203]. As TGF-β and HIF-1α have been demonstrated to exert synergistic effects to facilitate tumor progression as discussed in this review (summarized in Figure 4 and Figure 5, Table 1), it would be of interest to combine inhibitors targeting TGF-β signaling through TGFβRI with drugs such as Galunisertib [204], a TGFβRI kinase inhibitor with drugs that specifically are targeting HIF-1 [200]. An interesting and specific drug which specifically is targeting HIF-1 and HIF-2 is acriflavine which by binding directly to HIFs can counteract dimerization of HIF-1 and thereby its transcriptional activity [205] has been shown to inhibit growth of prostate cancer and colorectal cancer in preclinical models [206].

Acriflavine has been shown to inhibit growth of prostate cancer and colorectal cancer in preclinical models. Acriflavine is a heteroaromatic dye with antibacterial and antiviral effects [207] which also has been shown to inhibit TGF-β-induced EMT [208]. Notably, treatment with PT2385, a specific HIF-2a antagonist, was recently demonstrated to have favorable effects in patients with ccRCC with low toxicity showing promise for a first in class and specific HIF inhibitor in a clinical study [201]. A challenge for obtaining a successful treatment response with drugs targeting HIF-1 alone or in combination with other drugs is the distance between blood vessels in tumors and the hypoxic cancer cells (Figure 2), which could prevent the drug from being able to reach its target. 

Another possible treatment strategy could be to target HIFs and angiogenesis to prevent tumor progression. Treatment with acriflavine with sunitinib or digoxin was shown to reduce TGF-β expression in a murine breast cancer model and lung metastasis, respectively [209]. Since HIF-1 and TGF-β evoke synergistic effects to facilitate tumor progression in part by activating similar target genes, the usefulness of a combination of drugs that specifically inhibits HIF-1 and TGFβRI with treatment with drugs with a profile similar with Galunisertib should be further explored as a possible way forward in the development of more efficient treatment strategies in personalized medicine in the future. It should, however, be noted that treatment with general TGF-β inhibitors in clinical trials has not been as successful as expected, given that the results for preclinical cancer models have been very promising [210]. 

Moreover, and interestingly, HIF-1α and extracellular adenosine/A2 adenosine receptor-mediated immunosuppression is a physiologic response which offers protection of tissue damage induced during infections with antipathogen immune cells involved. Similar signaling pathways make tumor cells thrive in hypoxic tumors rich in extracellular adenosine and evolve resistance to our most common anti-cancer treatment drugs. Thus, a possible treatment strategy could be to induce hyperoxic conditions within these kinds of tumors by using specific oxygenation agents combined with specific inhibitors against HIFs as suggested by Hatfield and Sitkovsky in their recent review [199]. In a recent clinical study in treatment of patients with refractory ccRCC, adenosine A2A receptor blockade was demonstrated to show beneficial effects [211]. In this situation, it should also, in theory, be beneficial to inhibit the immunosuppressive effects induced by TGF-β [20].

In a recent study, ADAM17 was found to be overexpressed in liver fibrosis in a preclinical study of non-alcoholic steatohepatitis (NASH) [212]. ADAM17 has also been shown to be implicated in the proteolytic cleavage of TGFβRI, leading to aberrant TGF-β signaling in prostate cancer cells [47]. As ADAM17 plays an important and functional role in both cancer and fibrosis, it might be a potential drug target for treatment in these diseases, although the specificity is expected to be low, as ADAM17 is known to have a high number of substrates [213]. 

To facilitate development of novel, better and specific treatment strategies for tumors dependent on oncogenic TGF-β signaling pathways or HIFs, it is important to continue to search for novel drug targets and validate promising biomarkers to identify patients who would benefit from treatment with drugs targeting these pathways. Given the recent scientific progress within the field of personalized medicine, it is expected that, hopefully soon, we will see some of them be successfully translated into clinical use to benefit cancer patients.
